# *Ex vivo* study on prebiotic & choline combination to modulate gut bacteria, enhance choline bioavailability, and reduce TMA production

**DOI:** 10.20517/mrr.2024.90

**Published:** 2025-05-07

**Authors:** Ying Qi Goh, Guoxiang Cheam, Mingyue Yeong, Nidhi Bhayana, Abigail Thomson, Jingtao Zhang, Jia Xu, Patricia Conway, Smeeta Shrestha, Yulan Wang

**Affiliations:** ^1^Lee Kong Chian School of Medicine, Nanyang Technological University, Singapore 636921, Singapore.; ^2^School of Biological Sciences, Nanyang Technological University, Singapore 637551, Singapore.; ^3^Singapore Phenome Centre (SPC), Nanyang Technological University, Singapore 636921, Singapore.; ^4^Brenner Centre for Molecular Medicine, Singapore Institute for Clinical Sciences (SICS), A*STAR, Singapore 117609, Singapore.; ^5^Singapore Centre for Environmental Life Sciences Engineering, Nanyang Technological University, Singapore 637551, Singapore.; ^6^Centre for Marine Science and Innovation, School of Biological, Earth and Environmental Sciences, The University of New South Wales, Sydney, NSW 2052, Australia.; ^#^Authors contributed equally.

**Keywords:** Choline, trimethylamine lyase, prebiotics, gut, *Clostridium*, chorismate, tryptophan

## Abstract

**Aim:** Choline is a universal methyl group donor, playing an essential role in DNA methylation, signaling pathways, and the transport and metabolism of lipids. The primary source of choline intake is diet, and chronic deficiency has been associated with dementia, cardiovascular disease, and liver disease. Choline bioavailability can be diminished by gut microbes that express choline trimethylamine-lyase (*cutC*), an enzyme that converts choline into trimethylamine (TMA), a precursor for TMA N-oxide (TMAO), which is associated with an increased risk of cardiovascular diseases. Gut microbiota modulation can be achieved by prebiotics such as galactooligosaccharides, inulin, and fructooligosaccharides. The aim of our study is to use choline with prebiotics to modulate the gut microbiota to enhance choline bioavailability and minimize TMA production.

**Methods:** We employed an *ex vivo* microcosm system consisting of healthy human stool samples with choline and different prebiotics and measured TMA and choline levels by targeted metabolomics. Shotgun metagenomic profiling was also performed to investigate alternation in gut microbiota composition during choline and prebiotic interventions.

**Results:** Our study showed that choline to TMA conversion is dependent on a choline derivative and supplementing galactooligosaccharides (GOS) reduces this conversion. Choline to TMA conversion was associated with enriched microbiota from the genus *Dialister*, whereas GOS supplementation led to an increase in *Blautia* and a reduction in *Clostridia* populations. Loss of *Clostridia* also reduced a subset of *Clostridium* species, *Clostridium citroniae*, known to encode the *cutC* gene. The abundance of *Dialister* enhanced the chorismate biosynthesis pathway, while a reduction in *Clostridium* supported tryptophan and methionine pathways.

**Conclusion:** This study is the first to identify the combination of choline and GOS supplementation as a potential strategy to modulate gut microbiota and its metabolites in order to improve disease etiology.

## INTRODUCTION

Choline is an important nutrient integral to human metabolism and immensely impacts overall health. Found in both plant and animal tissues, choline intake is largely dependent on dietary sources^[1]^. Dietary choline is available in free form or as derivatives such as phosphocholine (PC), glycerophosphocholine (GPC), sphingomyelin (SM), or phosphatidylcholine (PtdCho)^[2]^. These forms of choline are vital for numerous biological processes, including neurotransmitter synthesis, cell membrane signalling, lipid transport, and methyl-group metabolism^[3]^ .

Recent reviews assessing dietary choline in European and non-European populations revealed inadequate choline intake among adults^[4]^. The pathological implications of a choline-deficient diet are significant, particularly given choline's role as a methyl donor. A reduced choline pool can impair the methylation of homocysteine to methionine, leading to elevated plasma homocysteine levels, which are associated with an increased risk of cardiovascular diseases, atherosclerosis, and stroke^[5]^. Additionally, methionine is converted into S-adenosylmethionine (SAM) by methionine adenosyltransferase, serving as a key methylating agent in various enzymatic methylation reactions throughout the body^[6]^. Prolonged choline deficiency can precipitate various pathological conditions, including muscle damage, liver damage, and non-alcoholic fatty liver disease^[7]^.

The intestinal microbiota play a pivotal role in nutrient harvesting and its modifying bioavailability. Trimethylamine (TMA) production is a key microbial process influenced by diet, with implications for health. It primarily arises from the microbial metabolism of dietary precursors like choline, L-carnitine, and betaine, found in red meat, eggs, dairy, fish, and certain plant-based foods like spinach and beets^[8]^. The gut bacteria can metabolize choline into TMA, which can then be converted to trimethylamine-N-oxide (TMAO) in the liver by flavin-containing monooxygenases (FMOs)^[9,10]^. Human studies have established that TMAO levels in serum are positively correlated with impaired renal function^[11]^, colorectal cancer^[12]^, and cardiovascular disease (CVD)^[13]^. Although there are some cohort-specific studies that failed to find an association between TMAO levels and CVD^[14]^, an animal study showing choline and betaine to promote atherosclerosis is very convincing.

*Desulfovibrio desulfuricans*, a common choline-degrading bacteria, is known to encode choline trimethylamine-lyase (*cutC*), a glycyl radical enzyme^[15]^, and its activating enzyme (*cutD*). These enzymes are involved in the conversion of choline to TMA^[16]^. The *cutC/cutD* gene cluster is prevalent within the gut microbiota and is found in bacteria belonging to the phyla Actinobacteria, Proteobacteria, and Firmicutes^[17]^. Gut colonization of TMA-producing bacteria can lower serum choline levels and increase *Lachnoclostridium* and *Clostridium* - key *cutC*-containing genera that have been observed in atherosclerosis patients compared to healthy individuals^[18]^. Reducing microbial conversion of choline to TMA is crucial for improving choline bioavailability while limiting TMA production. Studies suggest that free choline is more readily utilized by choline-metabolizing bacteria than choline-containing derivatives like phosphatidylcholine, indicating that the conversion rate may depend on the choline compound’s structure^[19]^. Therefore, evaluating the conversion rate of different choline-containing molecules by the gut microbiota could be instrumental in developing nutritional strategies to enhance choline intake while minimizing TMA production. Alternatively, modulating the gut microbiota through dietary fiber intake, particularly by promoting beneficial bacteria such as *Bifidobacterium*^[20]^, may help reduce TMA production^[21]^. Prebiotics such as galactooligosaccharides (GOS), inulin, and fructooligosaccharides (FOS) support gut health by fostering a balanced microbiome^[22]^ and regulating TMA production. They enhance the growth of beneficial bacteria while suppressing TMA-producing species such as *Escherichia coli* and *Clostridium sporogenes*^[23]^. High-fiber, prebiotic-rich diets, common in Mediterranean and other plant-based dietary patterns, contribute to lower TMAO levels compared to Western diets^[24]^. Given the microbiota’s influence on choline metabolism, prebiotics may help limit its conversion to TMA, a precursor to TMAO, which is associated with cardiovascular disease. Our study investigates various choline derivatives as substrates for TMA production, assesses the effectiveness of different prebiotics in reducing choline conversion to TMA, and evaluates their impact on gut microbiota composition and function. Our findings underscore the crucial role of prebiotics in shaping microbial communities and their metabolites through choline metabolism, potentially offering health benefits, particularly in cardiovascular disease prevention.

## METHODS

### Study participants and sample collection

A total of 28 participants aged 45-65 years were recruited for this study [Supplementary Table 1]. Inclusion criteria include body mass index (BMI) < 27.5, non-smoker, and antibiotic use within the three months prior to sampling. Exclusion criteria were individuals with diabetes, psychiatric disorders (e.g., major depression), neurological disorders, life-threatening diseases (e.g., cardiovascular diseases), and gastrointestinal diseases. The participant recruitment and sample collection were approved by the Nanyang Technological University Institutional Review Board (IRB-2018-08-022). Two days before stool collection, participants had to abstain from caffeine-containing substances and vigorous physical activities. Each participant provided a stool sample in an air-tight container, which was immediately processed (within an hour after defecation) in an anaerobic chamber. Approximately 20 g stool samples were transferred into sterile cryovials, frozen, and stored at -80 °C with the CO_2_ sachet.

### Human stool *ex vivo* system and metabolite assay

The *ex vivo* system was set up by preparing a 20% (w/v) human fecal slurry from individual subjects each, with 100 mL Wilkins-Chalgren (WC) anaerobic broth (Thermo Fisher) in an anaerobic chamber (Coy Laboratories, atmosphere of 95% nitrogen and 5% hydrogen). Homogenized 20% fecal slurry was dispensed into 15 mL Falcon tubes and mixed with an equal volume of WC broth containing 3mM of a single choline derivative: choline chloride (C_5_H_14_CINO), glycerophosphocholine (C_8_H_20_NO_6_P), Phosphocholine (C_5_H_15_NO_4_P+) (Sigma-Aldrich), L-*α*-Phosphatidylcholine (C_42_H_80_NO_8_P) (Sigma-Aldrich), and egg sphingomyelin (C_39_H_79_N_2_O_6_P) (AvantiA). WC broth without choline derivatives was added to control tubes. Then, the Falcon tubes were transferred into a 2.5 L Oxoid anaeroJar (Thermo Fisher) with an AnaeroGen Compact paper sachet (Thermo Fisher) to maintain the anaerobic condition and incubated on a rotator at 180 r.p.m for 24 h at 37 °C. All the experiments were carried out in technical triplicates. At each time point (0, 4, 8, and 24 h), samples were collected from the falcon tube inside the anaerobic chamber for NMR analysis and for reverse transcription polymerase chain reaction (RT-PCR) (0 and 8 h), and stored at -80 °C until further experiments. For prebiotic experiments, WC broth for the homogenized fecal slurry was added with 1% (w/v) prebiotics of either GOS (Oligomate 55NP, Yakult Pharmaceutical Industry Co., Ltd), FOS (Sigma-Aldrich), or inulin (Sigma-Aldrich). The subsequent procedure was followed as mentioned above. Each sample came from an individual subject. Samples were not pooled for any study.

### Culture and optical density measurement of desulfovibrio desulfuricans

*Desulfovibrio desulfuricans* ATCC 27774 was cultured in Tryptic Soy Medium (TSM, Merck, Singapore) supplemented with 5% defibrinated Sheep Blood (SB, Thermo Fischer Microbiology, Singapore) and incubated in an anaerobic chamber (BACTRON, US) at 37 °C for 96 h. After 96 h, the *Desulfovibrio desulfuricans* culture was inoculated into fresh TSM + SB medium and incubated for an additional 24 h. This overnight *Desulfovibrio desulfuricans* growth culture was inoculated into TSM + SB media and divided into four portions as (1) control; (2) with 3mM Choline Chloride (Sigma Aldrich, Singapore); (3) with 1% GOS (Yakult Pharmaceuticals, Japan); and (4) with 1% GOS + 3mM Choline Chloride, and incubated in an anaerobic chamber. After 72 h, the *Desulfovibrio desulfuricans* cultures were processed for nuclear magnetic resonance (NMR) and RT-PCR analysis. All treatment groups were carried out in technical triplicates. The *Desulfovibrio desulfuricans* culture was retrieved from an anaerobic chamber and 200 uL was used for OD600nm measurement using a 96-well ELISA plate reader. The absorbance values were normalized against blank (Tryptic Soy medium without added Defibrinated Sheep Blood).

### Quantification of metabolites using ^1^H-NMR

Microtubes containing *ex vivo* fermentation human stool samples from individual subjects were centrifuged at 16000 g, 4 °C for 10 min, after which 500 uL of supernatant were collected and added into 50 µL PB (150 µM K_2_HPO_4_ and NaH_2_PO_4_, 200 µM NaN3 99.9% D_2_O) containing internal standard Trimethylsilyl propanoic acid 0.1% m/v for NMR analysis. The NMR spectra were recorded on a 600 mHz Ascend NMR spectrometer (Bruker) equipped with a 5mm BBI Z-Gradient high-resolution probe. Samples were kept at 5 °C in the SampleJet autosampler, and the probe temperature was set at 300 K during the acquisition. A standard one-dimensional pulse sequence Noesypr1d was used with a 90-pulse length of approximately 11 μs (-11.03 dbW) on Bruker spectrometer. For each sample, the spectral width was 20ppm and 32 transients were collected into 65,536 data points. Receive gain was automatically determined. Water suppression was achieved with weak irradiation during the recycle delay (4 s) and mixing time (10 ms). An exponential window function with a line broadening factor of 0.3 Hz was applied to all the free induction decays before Fourier transformation. TopSpin (v4.0.9, Bruker) was used for spectra processing. The phase and baseline were corrected manually, and the chemical shift of TSP was calibrated at 0.00 ppm. The TMA peak (*δ* 2.86 to *δ* 2.90) was integrated from the normalized NMR spectra. Relative concentrations of TMA (C_i_-C_0_)/C_0_ were calculated.

### Quantification of cutC cDNA and DNA copy from human stool and Desulfovibrio desulfuricans samples

RNA was extracted from fecal samples using the Stool Total RNA Purification Kit (Norgen), following the manufacturer’s instructions. Extracted RNA was treated with Rnase-free-DNase1 (Thermo Fisher) and reverse-transcribed to cDNA using ReverTra Ace-α- (Toyobo). PCR was then performed using endogenous 16s rRNA as an internal control for relative quantification. The oligonucleotide primer sequences used for PCR were as follows: *cutC* forward 5’-TTYGCIGGITAYCARCCNTT-3’ and reverse 5’-TGNGGYTCIACRCAICCCAT-3’ and 16s rRNA forward 5’AGRGTTHGATYMTGGCTCAG-3’ and reverse 5’-TGCTGCCTCCCGTAGGAGT-3’ (Integrated DNA Technologies). Cultures of *D. desulfuricans* were centrifuged and the resulting bacterial pellets were processed using the GenElute^TM^ Bacterial Genomic DNA kit (Sigma Aldrich, Singapore) as per the manufacturer’s instructions. DNA quantified for cutC gene copy number via quantitative PCR using a standard curve from serially diluted plasmid pET-28a-*cutC*^[25]^. The abundance of cutC gene copy number was expressed as a percentage relative to the *16s rRNA* gene copy number. The oligonucleotide primer sequences used for PCR were the same as above.

### Shotgun metagenomic sequencing and microbiome analysis

Shotgun metagenomic sequencing of human stool samples from a single individual subject was conducted by BGI (Shenzhen, China), with subsequent bioinformatics and biostatistics analyses carried out in-house. Sample details are as follows - Individual subjects were divided into following groups, (a) control, AEF02, AEF03, AEM05, AHF01, AHF04, AHM01, AHMO6A; (b) choline, AEF02-Choline, AEF02-GPC, AEF02- PC, AHF01-Choline, AHF01-GPC, AHF01-PC, AHM01-Choline; and (c) choline + GOS, AEF02G-Choline, AEF02G-GPC, AEF02G-PC, AHF01G-Choline, AHF01G-GPC, AHF01G-PC, AHM01G-Choline. A total of 21 samples from 11 individual subjects were sequenced. Quality assessment of the raw sequencing reads was conducted using FastQC^[26]^, followed by adaptor and read quality trimming with BBDuk^[27]^. Sequence decontamination was performed using Homo sapiens NCBI GRCh38 reference genome with Bowtie2^[28]^. Taxonomic and functional annotations were obtained with Metaphlan^[29]^ and HUMAnN 3.9^[30]^, respectively. The reads were annotated to genes (Uniref90) and pathways using HUMAnN 3.9. Statistical analysis was performed using MaAsLin2^[31]^ after normalizing sequence reads to relative abundance. Data visualization, as well as alpha and beta diversity analysis and principal component analysis, was performed using R version 4.3.1^[32]^ and the MicrobiomeAnalyst software^[33]^. Taxonomy correlation network analysis was performed using NetCoMi^[34]^ in R version 4.3.1. For single network analysis, the netconstruct function was used with the following parameters: measure = “pearson”, filtTax = “highestFreq”, filtTaxPar = list (highestFreq = 50), zeroMethod = “pseudo”, zeroPar = list (pseudocount = 0.5), normMethod = “clr”, sparsMethod = “threshold”, thresh = 0.8). Only taxa that passed a t-test with a significance level of 0.05 were visualized in the network. The fast greedy clustering method was used to illustrate the community structure.

### Statistical analysis

All sample metabolite levels and *cutC* gene copy number data were analyzed using Student’s *t*-test in GraphPad Prism v9. All data had a minimum of three biological replicates and three technical replicates. Microbiome abundance was expressed as the standard deviation (SD), while variability in transcript and gene copy numbers was expressed as the standard error of the mean (SEM), with *P* value < 0.05 considered statistically significant.

## RESULTS

### Microbial TMA production is substrate-specific

**Figure 1:**
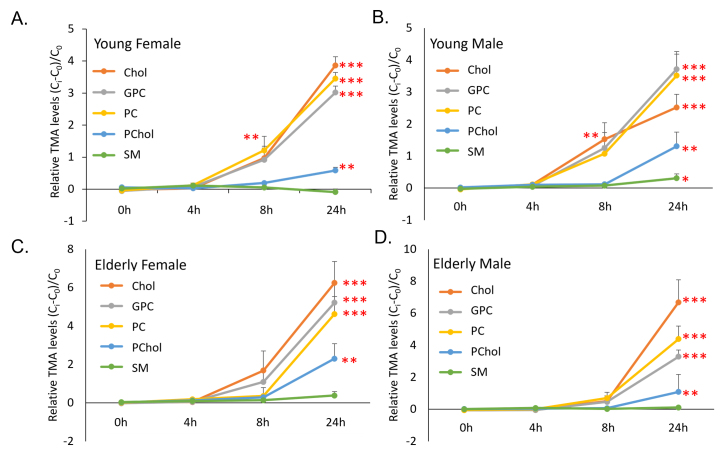
Microbial TMA production is substrate-specific. *In-vitro* anaerobic fermentation of human stool with different choline derivatives was performed, and relative TMA levels were measured by NMR analysis. Trendlines illustrating the TMA conversion levels of different substrates at time points 0 h, 4 h, 8 h, and 24 h across (A) young female (*n* = 4); (B) young male (*n* = 5); (C) elderly female (*n* = 4); and (D) elderly male subjects (*n* = 5) were plotted. Choline (Cho) and its derivatives Glycerophosphocholine (GPC), Phosphoric choline (PC), phosphatidylcholine (Pchol), and sphingomyelin (SM) were added to the *ex vivo* fermentations. C_0_ - concentration at time 0, C_i_ - concentration at individual time point. A *t*-test was performed to compare TMA production from choline derivatives at individual time points. Data are presented as mean ± standard deviation. ***: *P* value < 0.001, **: *P* value < 0.01, *: *P* value < 0.05. All experiments were performed in triplicates with individual subjects. TMA: trimethylamine; NMR: nuclear magnetic resonance.

To investigate the conversion of TMA from choline and its derivatives across gender and age, an *ex vivo* anaerobic fermentation of human stool obtained from young (45-55 y, Figure 1A and B) and elderly (> 55 y, Figure 1C and D) subjects from both genders with different choline derivatives were analyzed. Choline (Cho), GPC, and PC showed higher rates of TMA conversion, whereas phosphatidylcholine (Pcho) and SM exhibited slower conversion rates [Figure 1A-D]. A comparative analysis of relative TMA levels at 24 h revealed age-related differences in response to various choline substrates. Specifically, female subjects in the younger group exhibited significantly lower TMA levels than their elderly counterparts across choline, GPC, PC, and PCho [Supplementary Figure 1A]. In contrast, among males, this significant age-related difference was observed only with choline [Supplementary Figure 1B].

### Choline-mediated increase in TMA was significantly reduced by GOS

**Figure 2:**
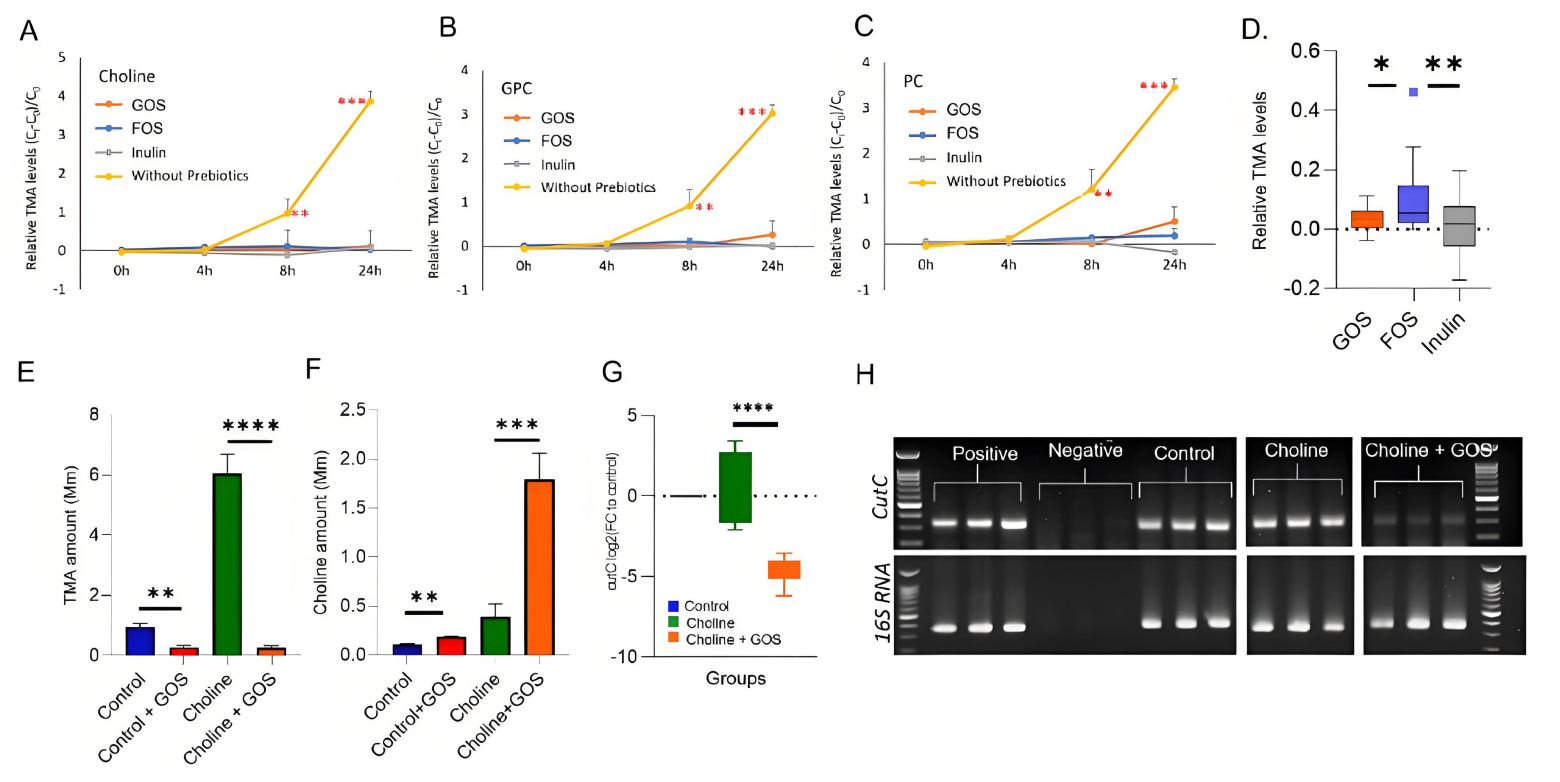
Choline-mediated increase in TMA was significantly reduced by GOS. Trendlines illustrating relative TMA conversion levels on treatment with (A) choline; (B) glycerophosphocholine; and (C) phosphoric choline across 0 h, 4 h, 8 h, and 24 h time points with different prebiotics (GOS, FOS, inulin). Whisker plot showing relative TMA levels across GOS (G); FOS (F); and inulin (I). Bar plot showing (E) TMA and (F) choline levels across control, control + GOS, choline, and choline + GOS. The *ex vivo* cultures include control (without prebiotics), choline, and choline + GOS. RNA was extracted, converted to cDNA, and analyzed by real-time quantitative PCR (qPCR) to quantitate (G) the transcript levels of *cutC* relative to the control. The qPCR product was run on 1X tris acetate ethylenediaminetetraacetic acid (TAE)gel in the following order: ladder, positive control (*Desulfovibrio desulfuricans*), negative control (no template control), control, choline, and choline + GOS. Student’s *t*-test was used to analyze the TMA, choline, and *cutC* levels, with data shown as mean ± SEM. ***: *P* value < 0.001, **: *P* value < 0.01, *: *P* value < 0.05 compared to respective controls. Each data point in the analysis represented an individual subject (*n* = 4) and all experiments were performed in triplicates. C_0_ denotes the concentration at 0 time point and C_i_ indicates the concentration of metabolites at individual time point. *cutC*: choline trimethylamine-lyase; TMA: trimethylamine; GOS: galactooligosaccharides; SEM: standard error of the mean.

To investigate the effect of the prebiotic supplement on the microbial conversion of choline derivatives to TMA, the stool samples from participants were incubated with TMA-producing choline derivatives (choline, PC, and GPC) in different prebiotics-supplemented media, namely GOS, FOS, and inulin. A time series graph measuring relative TMA levels showed that all three prebiotics suppressed the conversion of choline derivatives to TMA [Figure 2A-C]. On comparison of relative TMA levels across the three prebiotics, GOS significantly reduced TMA with the lowest variance [Figure 2D]. GOS demonstrated a strong inhibition of choline conversion to TMA and was selected for further experiments.

Human stool samples from individual subjects were cultured as control (without prebiotics), control + GOS, choline, and choline + GOS, and the amount of TMA and choline was quantified after 24 h. We observed a significant reduction in TMA [Figure 2E] and an increase in choline[Figure 2F] in the choline + GOS group. This significant reduction in TMA and availability of choline indicates a long-lasting inhibition of *cutC* activity, possibly achieved via suppression of TMA producers at the community level or the inhibition of *cutC* expression at the genomic level. The *cutC* expression level was then measured by RT-PCR and a significant reduction in *cutC* was observed in the choline + GOS group (compared to control) [Figure 2G and H], which can explain the reduced TMA levels.

### Enrichment of genus Blautia and reduction in Clostridium in the choline + GOS group

**Figure 3:**
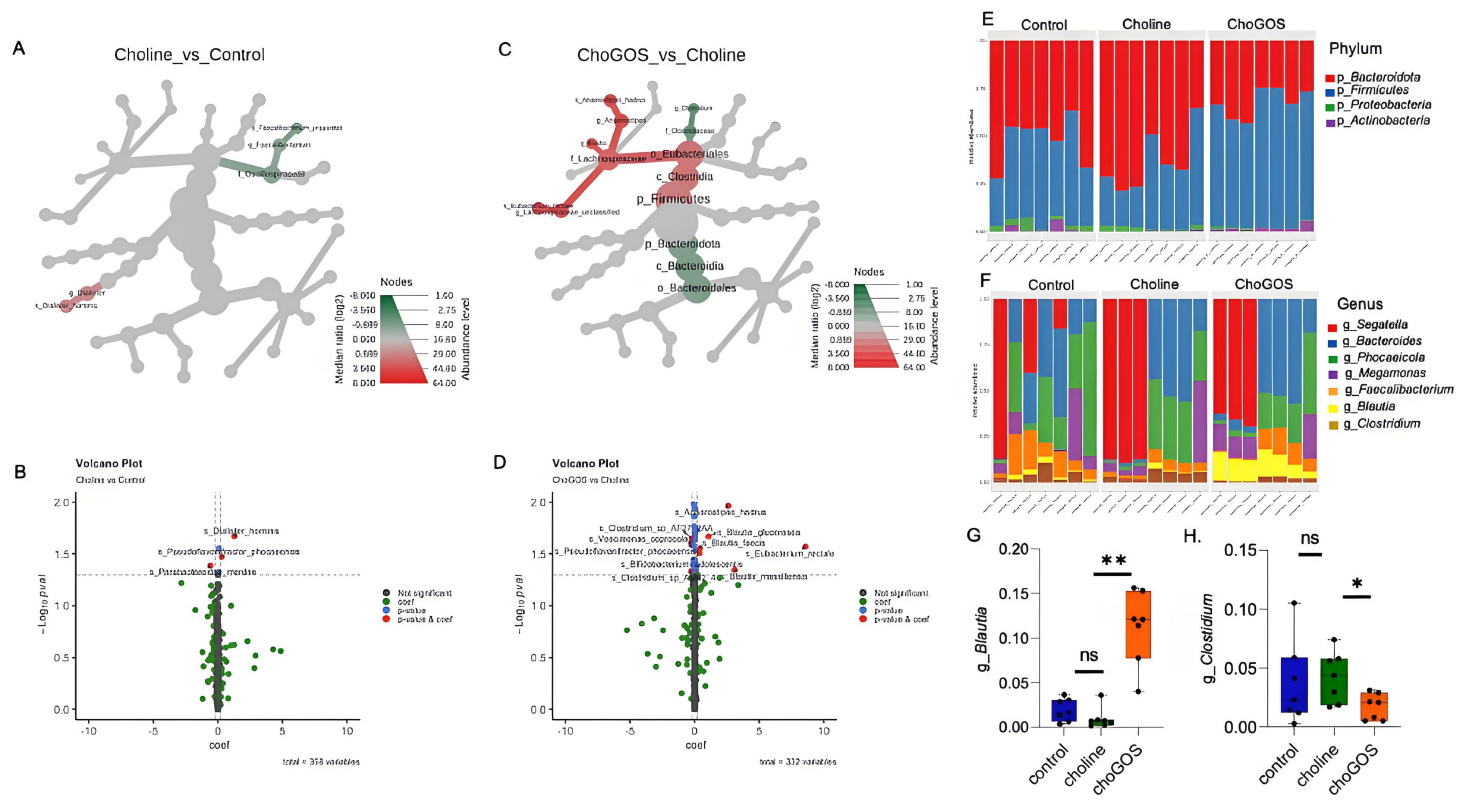
Enrichment of *Blautia* and reduction in *Clostridium* in the choline + GOS group. The hierarchical heat tree illustrates microbial abundance differences between (A) control *vs.* choline and (C) choline *vs.* ChoGOS. Red and green colors indicate higher and lower abundances, respectively, highlighting distinct microbial profiles between the choline and ChoGOS groups. (B) and (D) Volcano plots showing significantly different species differences (*P* value < 0.05, effect size > 0.2) identified using MaAsLin2, (E) and (F) presenting the relative abundances at the phyla and genera levels, respectively, while (G) and (H) showing significant changes in the genera *Blautia* and *Clostridium* in the ChoGOS group, with statistical significance denoted as *** (*P* value < 0.001), ** (*P* value < 0.01), * (*P* value < 0.05). Control (*n* = 7), choline (*n* = 4), and choline + GOS (*n* = 4). Only microbial taxa with significant differences based on *t*-tests (*P* value < 0.05) are shown. Cho: choline; GOS: galactooligosaccharides.

To examine the microbiota profile in human stool samples, shotgun metagenomic sequencing was performed on *ex vivo* anaerobic cultures. The subjects (*n* = 11) were divided into three groups: control (*n* = 7), choline (*n* = 4), and choline + GOS (*n* = 4). The term “choline” collectively refers to “choline and its derivatives (GPC, PC)”. Taxa abundance was analyzed first between the Choline and Control groups, and second between the ChoGOS and Choline groups. Heat tree analysis revealed a notable reduction in *Oscillospiraceae* and an increase in *Dialister* [Figure 3A] in the choline group compared to the control. The choline group also showed a significant increase in the species *Dialister hominis*, *Pseudoflavonifractor phocaeensis*, and a reduction in *Parabatceroides merdae* [Figure 3B]. Compared to the choline group, the ChoGOS group displayed a significant increase in Firmicutes and a decrease in Bacteroidota. As per the tree, there was a significant increase in the family *Lachnospiracease* but a decrease in the family *Clostridaceae* [Figure 3C]. At the species level, ChoGOS showed a significant increase in *Anaerostripes hadrus*, *Blautia glucerasea*, *Eubacterium rectale*, and *Bifidobacterium adolescentis* and a reduction in *Clostridium sp AF27_2A* and *Verscimonas coprocola* [Figure 3D]. Further analysis of the relative abundance of taxa across the three groups at the phylum [Figure 3E] and genus levels [Figure 3F] revealed a significant increase in the genus *Blautia* [Figure 3G] and a reduction in *Clostridium* [Figure 3H] in the ChoGOS group across all samples.

### Reduction in Clostridium species encoding cutC and cutD in the choline + GOS group

A significant reduction in the genus *Clostridium* was observed in the choline + GOS group. Previous studies have identified *Clostridium* as a TMA producer that encodes the *cutC* gene^[16]^. To confirm this, we investigated the abundance of specific *Clostridium* species in our dataset. Notably, we found a reduction in *Clostridium citroniae, Clostridium fessum, Clostridium sp AF27_2AA, clostridium lavalense, Clostridium bolteas, Clostridium aldenese* in the ChoGOS group compared to the Choline-only group [Supplementary Figure 2A]. We then annotated the metagenome data using uniref90 to identify bacterial species encoding *cutC* and *cutD*, and to assess their relative abundance across the three groups. In the ChoGOS group, *Clostridium citroniae* strains encoding *cutC* [Supplementary Figure 2B] and *cutD* [Supplementary Figure 2C] were absent compared to the choline-only group.

### Altered microbiota diversity and distinct correlation networks across the three groups

Gut microbial diversity and complexity are key to understanding community structure and function. We analyzed alpha and beta diversity, as well as the microbial correlation networks, across the three experimental groups. The Shannon diversity index (H) was used to assess microbial species diversity within the communities. ANOVA revealed no statistically significant differences in alpha diversity among the three groups across all features (*F* = 2.89, *P* value = 0.08). Post hoc two-group comparisons using Welch’s *t*-test showed marginal differences between the control *vs*. choline groups (*t* = 2.07, *P* value = 0.06) and no significant difference between the ChoGOS vs choline groups (*t* = -0.85, *P* value = 0.409) [Supplementary Figure 3A]. Beta diversity was measured using Bray-Curtis dissimilarity to measure microbial differences between samples. Permutational multivariate analysis of variance (PERMANOVA) indicated a significant difference in beta diversity across the three groups (*F* = 2.9, *P* value = 0.026, R2 = 0.24) [Supplementary Figure 3B]. To further explore group-specific microbial interactions, we constructed microbial correlation networks based on Pearson correlation of microbial abundances. The control group exhibited a sparse correlation network [Supplementary Figure 3C], whereas the choline [Supplementary Figure 2D] and choline + GOS groups [Supplementary Figure 3E] showed denser and more clustered correlation patterns.

### Significant enrichment in Chorismate and reduction in the tryptophan biosynthesis pathway

**Figure 4:**
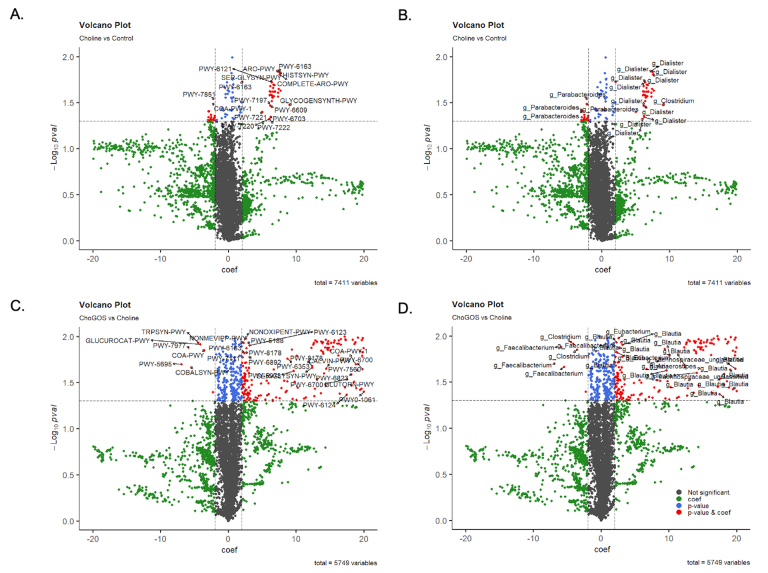
Significant enrichment in Chorismate and reduction in the tryptophan biosynthesis pathway. Volcano plots illustrate significantly altered metabolic pathways and their associated genera between choline and control groups (A and B), and ChoGOS versus choline (C and D). Microbial pathway abundance in the community was profiled using the HUMAnN pipeline, and differential analysis was performed using MaAsLin2 with a general linear model (feature ~ group). Volcano plots were generated in R. Red: *P* value < 0.02, coefficient > 2; Blue: P value < 0.02; Green: coefficient > 2. Black: not significant. Cho: choline; GOS: galactooligosaccharides.

We compared pathway enrichment between the choline and control groups and observed an increase in the superpathway of aromatic amino acid biosynthesis (COMPLETE-ARO-PWY), chorismate biosynthesis from 3-dehydroquinate (PWY-6163), and chorismate biosynthesis I (ARO-PWY) [Figure 4A]. This increase was associated with an increase in the genus *Dialister* [Figure 4B].

Next, we compared the ChoGOS group with the choline group [Figure 4C] and observed a significant reduction in L-tryptophan biosynthesis (TRPSYN-PWY), L-methionine biosynthesis IV (PWY-7977), and the superpathway of adenosylcobalamin salvage (COBALSYN-PWY), which corresponded to a decrease in the abundance of *Faecalibacterium* and *Clostridium* [Figure 4D].

Enriched pathways included the pentose phosphate pathway (PWY-8178) (non-oxidative branch), the Calvin-Benson-Bassham cycle (CALVIN-PWY), and various co-factor biosynthesis pathway required for the growth and function of *Blautia* [Figure 4D] The volcano plot highlights only those pathways and bacteria taxa with a *P* value < 0.05 and an effect size coefficient > 2. We also observed that, in the presence of added choline, *clostridium sp*. were enriched, and tryptophan biosynthesis was upregulated [Supplementary Figure 4A]. In the ChoGOS group, tryptophan biosynthesis was enriched primarily by *Blautia, Anaerostipes,* and *Lachnospiracease* [Supplementary Figure 4A and B]. A detailed annotation of all pathways is provided in Supplementary Table 2.

### ChoGOS significantly reduces Desulfovibrio desulfuricans growth and cutC gene copy number

**Figure 5:**
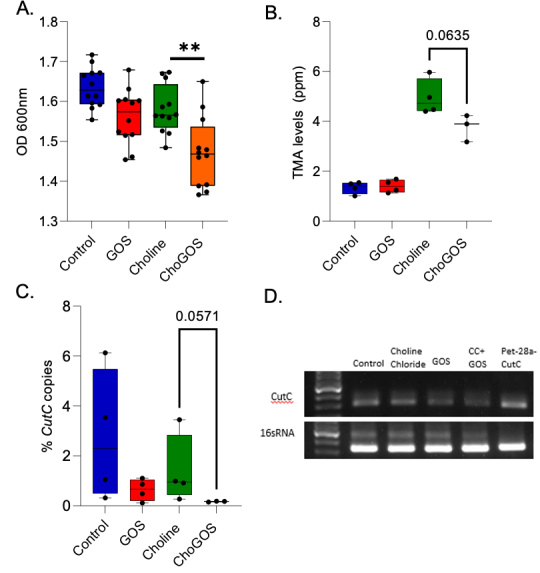
GOS inhibits *Desulfovibrio desulfuricans* growth and reduces *cutC* gene copy number*.* (A) Average optical density (OD) at 600 nm measurements show a significant reduction in *Desulfovibrio desulfuricans* growth in the ChoGOS group compared to the choline-alone group; (B) Box plot of the TMA concentrations in the culture media indicating a marginal reduction in TMA levels in the ChoGOS group (*P* value = 0.0635); (C) Box plot showing the percentage reduction in *cutC* gene copy number in the ChoGOS group (*P* value = 0.057); (D) Image of a 1.5% agarose gel (1x TAE) illustrating amplification of the *cutC* gene across different groups and a positive control, plasmid. Statistical comparisons between groups were performed using *t*-tests, and data are presented as mean ± SEM. Experiments were conducted in triplicate for each group: control, choline chloride (CC), choline + GOS, and CC + GOS, with *n* = 4 per group. Significance is noted as **: *P* value < 0.01. GOS: galactooligosaccharides; TMA: trimethylamine; Cho: choline; SEM: standard error of the mean; TAE: tris acetate ethylenediaminetetraacetic acid.

To determine whether GOS directly suppresses *cutC* expression or indirectly affects it by inhibiting the growth of *cutC*-encoding microbes, we used *Desulfovibrio desulfuricans (*ATCC 27774)*,* a choline-degrading, sulfate-reducing bacterium that carries the *cutC* gene^[25]^. A significant reduction in *D. desulfuricans* growth was observed in the presence of Choline + GOS compared to choline alone [Figure 5A]. This growth inhibition was accompanied by reduced TMA levels [Figure 5B]. We further quantitated the copy numbers of the *cutC* gene and 16s rRNA using RT-PCR with a standard curve method. The ChoGOS group showed a reduced percentage of *cutC* gene copies [Figure 5C], as well as diminished band intensity for the *cutC* gene [Figure 5D]. These findings indicate that ChoGOS may inhibit the growth of *cutC-*encoding gut microbes, leading to a concurrent reduction in *cutC* copy number and, consequently, TMA levels.

## DISCUSSION

Choline is an essential micronutrient, yet many individuals do not meet the recommended daily intake. Chronic choline deficiency has been associated with cognitive decline, liver and muscle damage, and elevated homocysteine levels - a known risk factor for CVD^[35]^. The conversion of dietary choline to TMA by the gut microbiota can further contribute to choline deficiency and its related cardiometabolic risks. In this study, we investigated gut microbiota-mediated choline metabolism and explored the use of prebiotics to modulate the microbiota in order to enhance choline bioavailability and minimize TMA production. Our findings suggest age, gender, and the specific form of choline consumed may influence its conversion to TMA. This insight could inform the development of personalized nutritional guidelines. Additionally, we observed that not all choline-containing derivatives are converted to TMA at the same rate by the gut microbiota. This indicates that different choline derivatives vary in their accessibility to microbial enzymes responsible for TMA production. For example, comparative studies have shown that consumption of phosphatidylcholine, common in eggs, results in lower plasma TMAO levels than intake of choline bitartrate, a common dietary supplement^[36]^. It is possible that the steric hindrance caused by the fatty acid chains in phosphatidylcholine impedes the phosphocholine head group from binding to the catalytic site of *cutC*. These findings may contribute to more effective dietary recommendations by guiding the selection of choline sources that minimize TMA generation and potentially reduce the risk of cardiometabolic diseases.

We further investigated the ability of different prebiotics to suppress microbially mediated choline metabolism to TMA and found that GOS exhibited the best performance compared to inulin and FOS. GOS, prebiotics produced through *β*-galactosidase transgalactosylation, selectively stimulate the growth of beneficial bacteria such as *Bifidobacterium*^[37]^*, Ruminococcus gnavus*^[38]^, and *Lactobacillus*^[39]^, which produce short-chain fatty acids that can reduce potential pathogenic bacteria^[40]^. In our study, we also observed an increase in *Bifidobacterium, Blautia, Lactobacilli*, and *Ruminococcus*, accompanied by a simultaneous reduction in *Coprobacter* and *Enterocloster.* The genus *Blautia* is an anaerobic bacteria widely distributed in the mammalian gut and has been reported to increase in abundance following supplementation with the prebiotic 2’-fucosyllactose^[41]^. Metagenomic studies have shown that individuals with increased levels of *Blautia* exhibit a proliferation of genes encoding extracellular α-l-fucosidase, an enzyme that releases lactose and fucose - both of which serve as substrates for *Blautia* growth. Studies have also reported that an increase in *Bifidobacterium* in the gut can antagonize the activity of spoilage bacteria such as *Clostridium sp*, thereby reducing the production of toxic fermentation byproducts. Additionally, a rise in *Blautia* abundance has been associated with decreased *Clostridium* colonization^[42]^. These observations support our findings that increases in *Bifidobacterium* and *Blautia* are linked to reductions in *Clostridium* species. Notably, the reduction in *Clostridium* observed with on GOS supplementation was particularly associated with the decrease in a subset of *Clostridium* species harboring genes for *cutC* and *cutD*.

Gut bacteria can metabolize choline into TMA, which is subsequently converted to TMAO in the liver. Bacterial families involved in this process include *Firmicutes* and *Proteobacteria*. Specifically, species such as *Bacteroides*, *Clostridium*, and members of the *Enterobacteriaceae* family have been identified as key players in choline metabolism^[13]^. Short-chain fatty acids (SCFAs) play a vital role in maintaining health and regulating disease by contributing to gut homeostasis^[43]^. Supplementation with choline has been shown to alter the gut microbiota, potentially modifying SCFAs and promoting an increase in the genus *Dialister*. Similarly, the presence of the prebiotics GOS has been associated with an increase in *Blautia* and a decrease in a subset of *Clostridium. Clostridium* species can utilize large amounts of nutrients that cannot be digested by host enzymes, leading to the production of numerous SCFAs that support intestinal homeostasis^[44]^.

In our investigation of prebiotics-mediated choline metabolism, we observed that choline stimulated TMA producers, which in turn promoted an increase in *Dialister*, enriching the chorismate pathway. In contrast, prebiotics led to a reduction in *Clostridium*, thereby enhancing the tryptophan pathway. Collectively, these findings suggest that choline metabolism induces shifts in gut microbiota that influence the shikimate pathway, one-carbon metabolism, amino acid metabolism, and immune modulation^[45]^. Choline is a key contributor to one-carbon metabolism, which plays a role in methyl group synthesis and amino acid metabolism^[6]^. Chorismate is an important intermediate in the shikimate pathway, responsible for the bacterial synthesis of aromatic amino acids such as tryptophan, phenylalanine, and tyrosine^[46]^. Choline has recognized anti-inflammatory properties, while chorismate-derived metabolites - such as tryptophan derivatives - can modulate immune responses^[47]^.

This study has two main limitations: first, the small sample size; and second, the use of an *ex vivo* model rather than a dietary intervention study involving choline + GOS in human subjects.

In summary, our study presents several key findings. First, microbial choline metabolism to TMA is influenced by the availability of specific substrates in the presence of prebiotics; Second, choline-induced TMA production was significantly reduced by GOS; Third, this reduction in TMA was associated with notable changes in gut microbiota, particularly a loss of subsets of TMA producers; Lastly, TMA-stimulated microbiota favored amino acid metabolism, while GOS promoted tryptophan and methionine biosynthetic pathways. We conclude that prebiotic-mediated modulation of the microbiota to reduce TMA production would be impactful in reducing the risk of cardiovascular disease.

## DECLARATIONS

### Availability of data and materials

The data that support the findings of this study are available from NCBI under project number PRJNA1223686. Other raw data that support the findings of this study are available from the corresponding authors upon reasonable request.

### Copyright

© The Author(s) 2025.
