# Supplementary material for: Ex vivo study on prebiotic & choline combination to modulate gut bacteria, enhance choline bioavailability, and reduce TMA production
Source: Microbiome Res Rep. 2025 May 7;4(2):21. doi: 10.20517/mrr.2024.90 (PMC12370400; doi:10.20517/mrr.2024.90)
Supplement: Supplementary file 1 [file mrr-4-2-21-SupplementaryMaterials.pdf]

Supplementary Material

Ex vivo study on prebiotic & choline combination to modulate gut bacteria, enhance choline bioavailability, and reduce TMA production.

Ying Qi Goh[1,#], Guoxiang Cheam[2,#], Mingyue Yeong[1], Nidhi Bhayana[1], Abigail Thomson[3], Jingtao Zhang[3], Jia Xu[4], Patricia Conway[5,6], Smeeta Shrestha[1], Yulan Wang[1,3]

[1]Lee Kong Chian School of Medicine, Nanyang Technological University, Singapore 636921, Singapore.
[2]School of Biological Sciences, Nanyang Technological University, Singapore 637551, Singapore.
[3]Singapore Phenome Centre (SPC), Nanyang Technological University, Singapore 636921, Singapore.
[4]Brenner Centre for Molecular Medicine, Singapore Institute for Clinical Sciences (SICS), A*STAR, Singapore 117609, Singapore.
[5]Singapore Centre for Environmental Life Sciences Engineering, Nanyang Technological University, Singapore 637551, Singapore.
[6]Centre for Marine Science and Innovation, School of Biological, Earth and Environmental Sciences, The University of New South Wales, Sydney, NSW 2052, Australia.
[#]Authors contributed equally.

[*]**Correspondence to:** Yulan Wang and Smeeta Shrestha; Lee Kong Chian School of Medicine, Nanyang Technological University, Singapore 636921, Singapore. E-mails: yulan.wang@ntu.edu.sg; smeeta.shrestha@ntu.edu.sg. ORCID 0000-0002-6560-4230

ORCID: Yulan Wang (0000-0002-2831-8737), Smeeta Shrestha (0000-0002-6560-4230)

Section S1: Tables

Subject	Gender	Age	Race	BMI Calculation	Have you suffered from any severe gastrointestinal

					diseases in the past 1 year?
AEM01	Male	69	Chinese	23.18	No
AEM02	Male	68	Chinese	22.84	No
AEM03	Male	66	Chinese	22.27	No
AEM04	Male	72	Chinese	23.80	No
AEF01	Female	71	Chinese	19.40	No
AEF02	Female	65	Chinese	26.52	No
AHF01	Female	55	Chinese	22.51	No
AHM01	Male	47	Indian	28.58	No
AHF02	Female	25	Indian	22.27	No
AHM02	Male	32	Chinese	27.44	No
AHF03	Female	56	Chinese	22.68	No
AEM05	Male	70	Chinese	22.84	No
AHM03	Male	40	Chinese	25.47	No
AHF05	Female	41	Indian	24.89	No
AEF03	Female	61	Chinese	20.20	No
AEM06	Male	69	Chinese	24.39	No
AHF04	Female	45	Chinese	19.31	No
AEM07	Male	69	Chinese	24.22	No
AHM04	Male	49	Chinese	26.29	No
AHF06	Female	55	Chinese	18.61	No
AEM08	Male	72	Indian	25.96	No
AEM09	Male	69	Chinese	26.56	No
AHM05	Male	50	Chinese	24.39	No
AHF07	Female	55	Chinese	0.00	No
AEF04	Female	69	Chinese	23.94	No
AHM06	Male	54	Chinese	28.31	No
AHM07	Male	50	Chinese	24.86	No
AEM10	Male	67	Chinese	19.49	No

Table S1. Demographics of participants involved in the various analysis conducted in the study.

Pathway ID	Pathway Name
PWY-6121	5-aminoimidazole ribonucleotide biosynthesis I
PWY-7851	coenzyme A biosynthesis II (eukaryotic)
COA-PWY-1	coenzyme A biosynthesis I (bacteria)
PWY-6163	chorismate biosynthesis from 3-dehydroquinate
PWY-7197	pyrimidine deoxyribonucleotide phosphorylation
SER-GLYSYN-PWY	superpathway of L-serine and glycine biosynthesis I
ARO-PWY	chorismate biosynthesis I
HISTSYN-PWY	L-histidine biosynthesis
COMPLETE-ARO-PWY	Super pathway of aromatic amino acid biosynthesis
GLYCOGENSYNTH-PWY	glycogen biosynthesis I (from ADP-D-Glucose)
PWY-6609	adenine and adenosine salvage III
PWY-6703	preQ0 biosynthesis
PWY-7221	guanosine ribonucleotides de novo biosynthesis
PWY7222	guanosine deoxyribonucleotides de novo
TRPSYN-PWY	L-tryptophan biosynthesis
GLUCUROCAT-PWY	superpathway of hexuronide and hexuronate ...
PWY-7977	L-methionine biosynthesis IV
COA-PWY	coenzyme A biosynthesis I (bacteria)
PWY-5695	inosine 5'-phosphate degradation
COBALSYN-PWY	Super pathway of adenosyl cobalamin salvage
NONMEVIPP-PWY	methylerythritol phosphate pathway I
PWY-7111	pyruvate fermentation to isobutanol (engineered)
NONOXIPENT-PWY	pentose phosphate pathway (non-oxidative)
PWY-5188	uroporphyrinogen-III I (from glutamate)
PWY-8178	pentose phosphate pathway (non-oxidative branch)
PWY-6892	thiazole biosynthesis I (facultative anaerobic bacteria)
SERSYN-PWY	L-serine biosynthesis
PWY-6353	purine nucleotides degradation II (aerobic)
PWY-6700	queuosine biosynthesis

PWY-6124	inosine-5'-phosphate biosynthesis II
PWY0-1061	superpathway of L-alanine biosynthesis
GLUTORN-PWY	L-ornithine biosynthesis I
PWY-7560	methylerythritol phosphate pathway II
CALVIN-PWY	Calvin-Benson-Bassham cycle

Table S2. Table shows the pathway terms and its full annotation. HUMAnN pipeline was used with MetaCyc database was used for pathway analysis.

Section S2: Figures

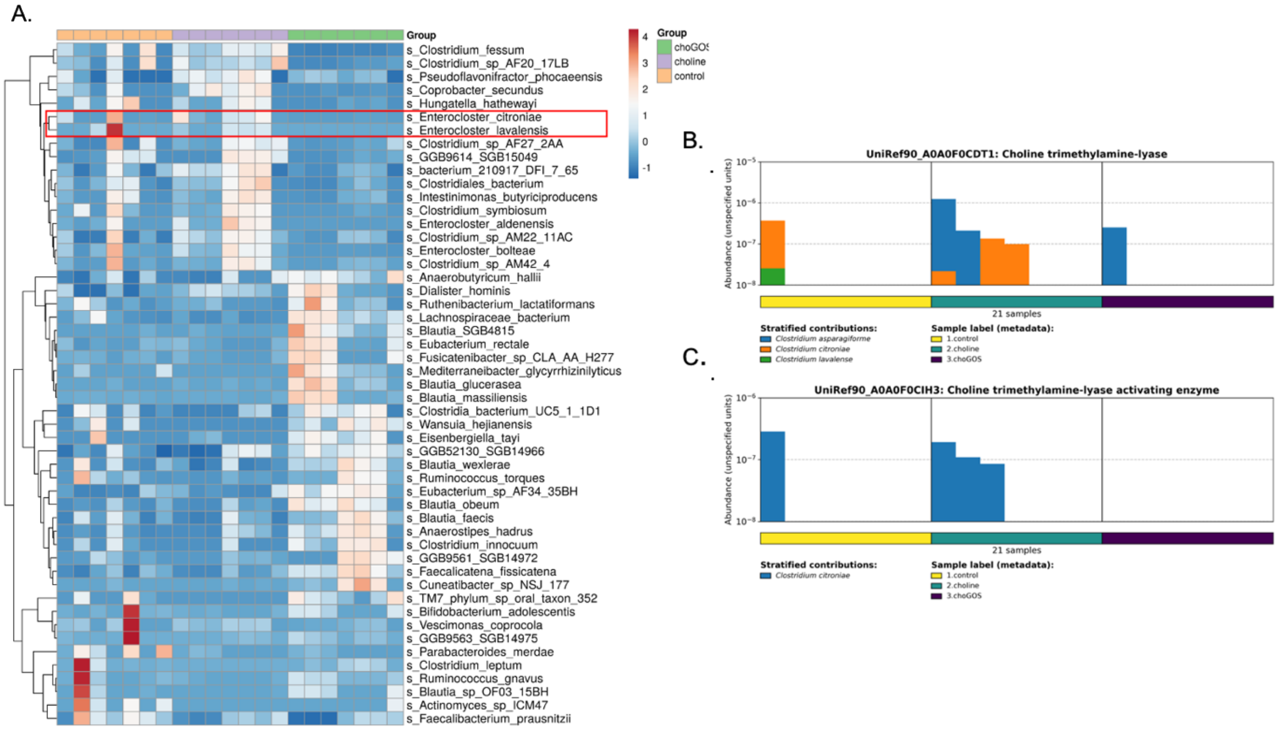

Figure S1. Reduction in Clostridium species encoding cutC and cutD in Choline + GOS group. (A) Heatmap showing list of bacteria species significantly different across the 3 groups. High abundance is indicated by red, and low abundance by blue. Heatmap shows only those pathway with significant difference at *P value* < 0.05.(B) Stratified bar plot showing abundance of *Clostridium* species which encode choline trimethylamine-lyase *(*cutC*)* and choline trimethylamine-lyase activating enzyme (CutD) gene across the 3 groups indicated in yellow (control), green (choline) and purple (choline+GOS).

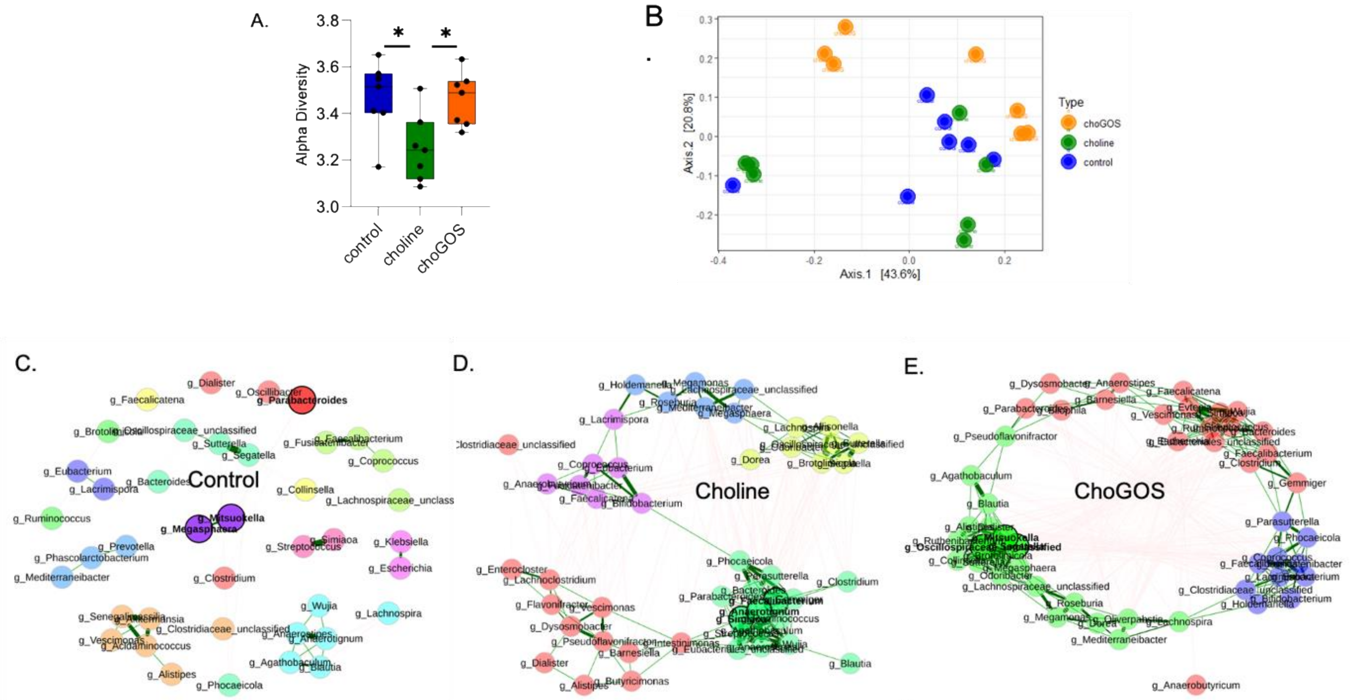

Supplementary Figure S2. Microbial diversity and correlation of microbiota in each group. (A) Shannon's alpha diversity among three dietary groups. (B) Microbial community differences across the three groups indicated by the Principal Coordinate Analysis (PCoA) based on Bray–Curtis dissimilarity distance. Single correlation network analysis at the genus level for (C) Control(D) Choline and (E) ChoGOS. NetCoMi , with netconstruct function with the following parameters, measure= "pearson", filtTax = "highestFreq", filtTaxPar = list(highestFreq = 50), zeroMethod = "pseudo", zeroPar = list(pseudocount = 0.5), normMethod = "clr", sparsMethod = "threshold", thresh = 0.8) was used to generate the correlation networks. Nodes represent genus, edges the correlation. Green edges indicate positive correlations and red edges indicate negative correlations, with edge thickness representing correlation strength.

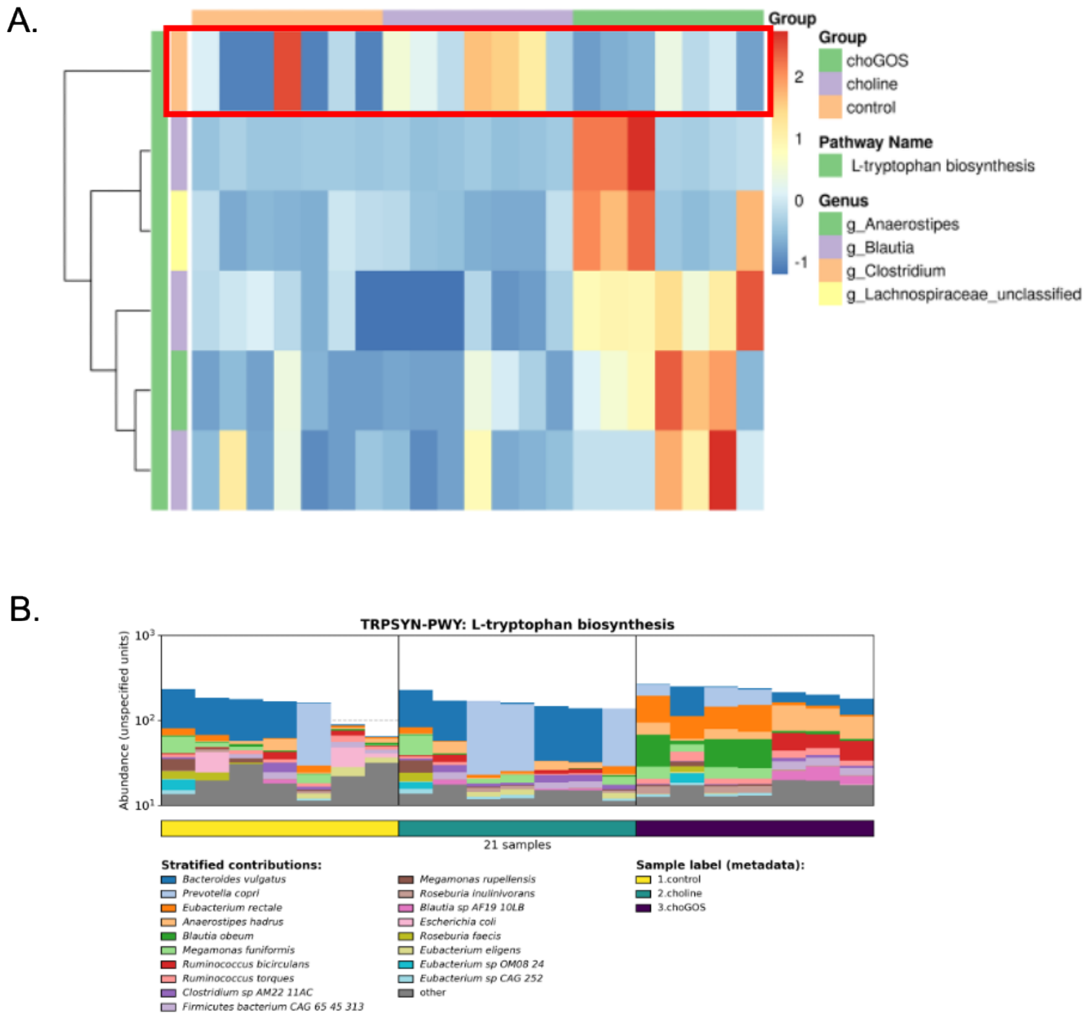

Figure S3. Microbiota- specific Tryptophan biosynthesis across 3 groups. (A) Heatmap illustrating the enrichment of different microbiota for tryptophan biosynthesis pathway across three groups. The red rectangle shows the pathways related to clostridium. The heatmap only includes pathways that show statistically significant differences ($p < 0.05$). (B) Stacked bar plot showing the stratified contribution of each microbe involved in the L-tryptophan biosynthesis pathway in each group.
